# The best surgical strategy for anal fistula based on a network meta-analysis

**DOI:** 10.18632/oncotarget.21836

**Published:** 2017-10-12

**Authors:** Qi Wang, Yukun He, Jun Shen

**Affiliations:** ^1^ The 1st Department of Gastrointestinal Surgery, Renmin Hospital of Wuhan University, Hubei Province, Wuhan 430060, P.R. China; ^2^ Department of General Surgery, Zhongnan Hospital of Wuhan University, Hubei Province, Wuhan 430071, P.R. China; ^3^ Emergency Center, Zhongnan Hospital of Wuhan University, Hubei Province, Wuhan 430071, P.R. China

**Keywords:** surgical strategy, anal fistula, network meta-analysis

## Abstract

**Objective:**

To determine a superior surgical treatment for anal fistula through a network meta-analysis and to provide the best direction for development in this field.

**Methods:**

We conducted a systematic literature search of the PubMed, Embase and Cochrane Library databases and extracted data from randomized controlled trials, which compared healing time, incontinence and recurrence associated with surgical strategies for anal fistula. A network meta-analysis was conducted using ADDIS software by evaluating the 3 parameters. Cumulative probability values were utilized to rank the strategies under examination. Inconsistencies were also tested using node-splitting models.

**Results:**

Twenty articles with 1663 patients were included. Fistulotomy plus marsupialisation had the shortest healing time (*P* = 0.69). Seton placement was the best procedure to avoid postoperative incontinence (*P* = 0.66). Fistulectomy exhibited the lowest recurrence rate (Probability *P* = 0.40). In general, fistulotomy plus marsupialisation and surgical ligation plus biomaterial plugging revealed superior clinical efficacy. Node-splitting model testing revealed that no significant inconsistency existed in this research.

**Conclusions:**

Fistulotomy plus marsupialisation exhibited preliminary superior surgical utility for anal fistula. Additionally, combination of surgical treatment with biomaterials may provide better clinical efficacy. These techniques may warrant consideration for future development in this field.

## INTRODUCTION

Anal fistula is a common disease in colorectal surgery with an incidence of 0.01% to 0.02% in the European population [1] and peak occurrence during the third and fourth decades of life [2]. Anal fistula is described as an abnormal tract connecting the anorectal mucosa to the exterior skin, which may present either de novo or after an acute anorectal abscess [3–4]. Anal fistulas are often associated with considerable inconvenience and morbidity. It is commonly believed that surgery is the only method of clinical cure. Although most cases are easily treated by various surgical options, the extended postoperative healing time, fecal incontinence and high recurrence rates still need to be ameliorated. As is well-known, the risk of postoperative recurrence ranges from 10% to as high as 57% [5]. Furthermore, repeated operations may lead to incontinence. Both repeated operations and incontinence may result in longer healing times. Thus, management of anal fistulas remains challenging.

Traditional surgical procedures include fistulotomy, fistulectomy, advancement flap, seton placement or fibrin glue fixation. Other treatments, such as “Biomaterial Plugging [6]” and “Marsupialization [7]”, were also invented to improve curative effects. Among these therapeutic approaches, each has its advantages. The experience of the surgeon may also have impact prognosis [8]. For example, biomaterial plugging may stimulate wound healing, although the quality of materials and different individual material requirements remain unpredictable factors. Simple marsupialisation may also confer potential benefits, but surgeon experiences and technique may also be the key to prognosis. Additionally, several new sphincter-preserving techniques have been developed and proposed recently, all with the common goal of minimizing anal sphincter injury and optimizing functional outcomes. However, the number of different procedures suggested, the lack of follow-up data and the often variable and conflicting clinical outcomes have generated confusion and skepticism, resulting in limited translation of these procedures into clinical practice [9].

Therefore, objectively speaking, the best surgical treatment for anal fistulas remains unclear. Given all of the facts above, based on current clinical therapeutic methods, it is important to find appropriate surgical treatments to simultaneously reduce healing time, fecal incontinence and recurrence rates. Accordingly, in this study, we aimed to determine the best surgical strategy for treatment of anal fistulas by conducting a network meta-analysis using the approaches currently regarded as the best tools for summarizing the extant scientific evidence.

## RESULTS

### Study characteristics and quality assessment

**Figure 1:**
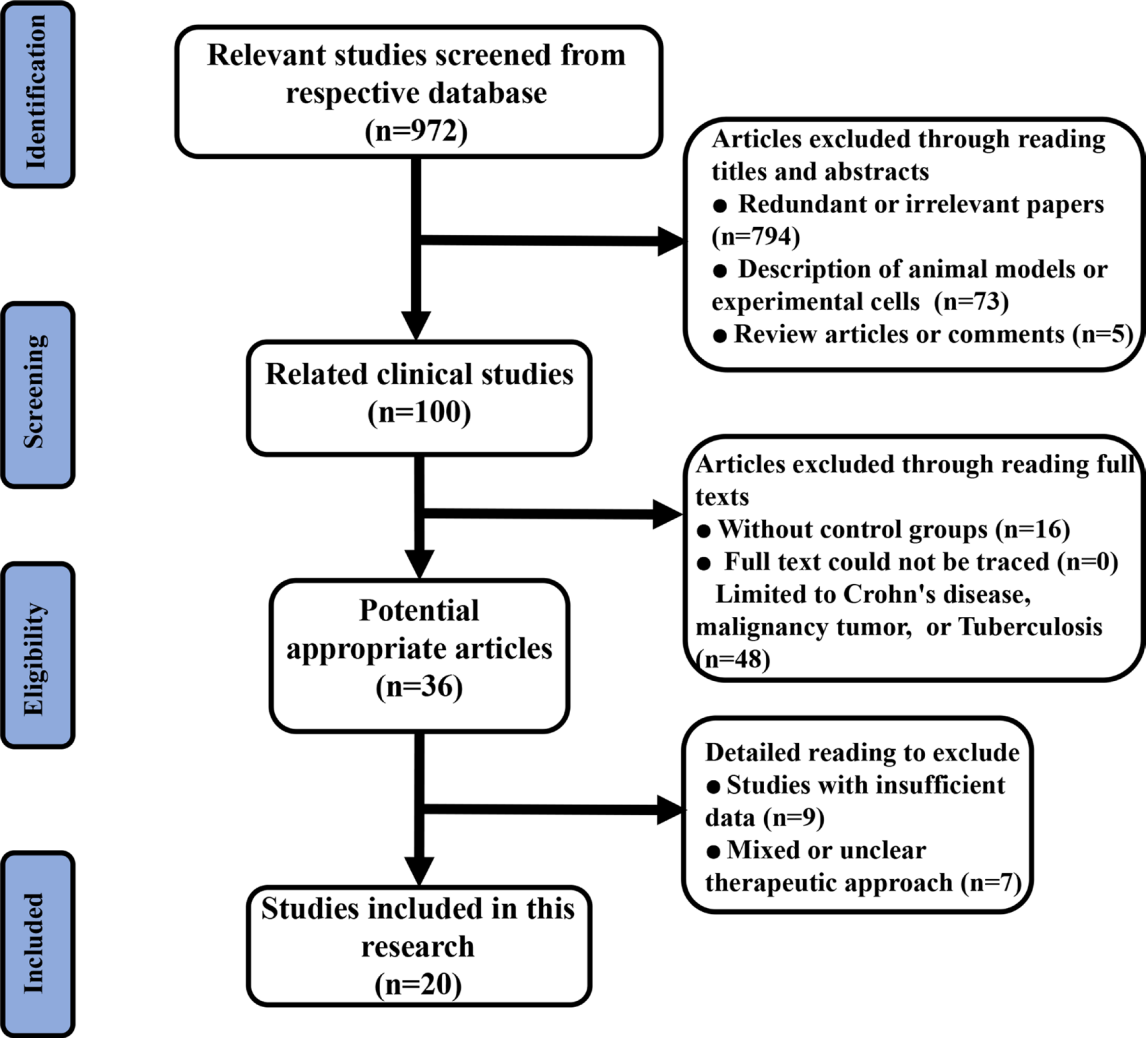
Flow diagram of the study selection process for this meta-analysis

**Figure 2:**
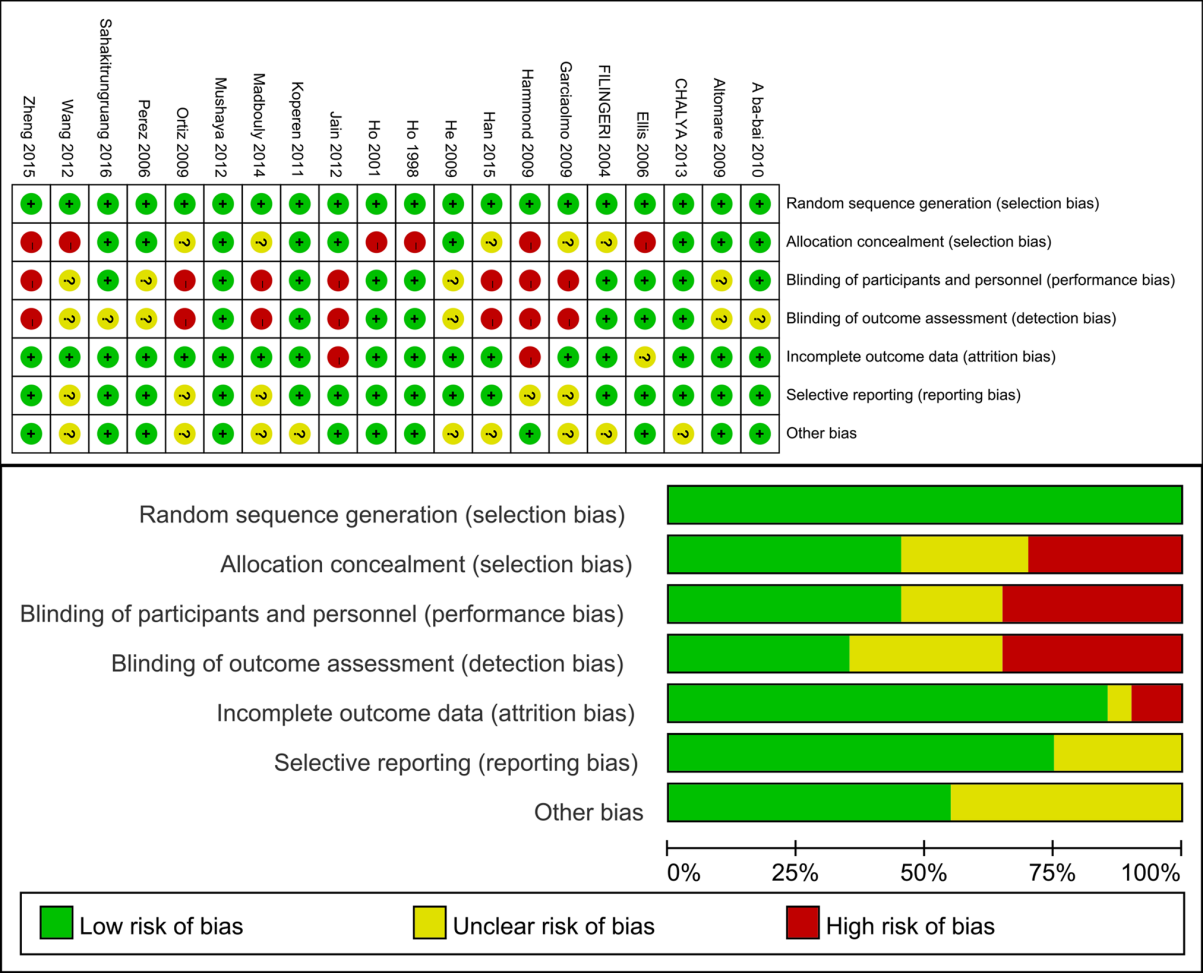
Methodological quality graph and summary of the included studies (top) Overall and (bottom) study-level risk of bias.

After detailed evaluation, 20 articles [10–29] including 1663 patients satisfied the study recruitment criteria (Figure 1). We included 20 RCTs which covered the usual surgical approaches, including Biomaterial Plugging (BP), Advancement Flap (AF), Seton Placement (SP), Fibrin Glue (FG), Fistulotomy, Fistulectomy, Marsupialisation, Surgical Ligation (SL), Fistula Plug (FP) and several combinations of these techniques. The characteristics of the included studies are presented in Table 1. Additionally, we addressed random sequence generation as reported in all of the included articles; a summary of the bias risk is shown in Figure 2. The overall quality of the included studies was good.

**Table 1:** Characteristics of the included trials

Author	Country	Pub. Year	Study Arms	Intervention	Included Sample Size (LF)	Follow-up Time	Parameter Data
A ba-bai [14]	China	2010	2	SL+BP vs. AF	90 (0)	5 months	Recurrence; Healing time; Faecal Incontinence
Altomare [15]	Italy	2009	2	SP vs. FG	62 (2)	12 months	Recurrence; Faecal Incontinence
CHALYA [16]	Tanzania	2013	2	Fo+Ms vs. Fe	162 (0)	12 weeks	Recurrence; Healing time; Faecal Incontinence
Ellis [17]	USA	2006	2	AF vs. AF+FG	58 (0)	36 months	Recurrence
FILINGERI [18]	Italy	2004	2	Fo vs. Fe	20 (2)	6 months	Recurrence; Healing time; Faecal Incontinence
Garciaolmo [19]	Spain	2009	2	BP+FG vs. FG	49 (1)	12 months	Recurrence;
Hammond [20]	UK	2009	2	BP+FG vs.BP	28 (1)	18 months	Recurrence;
Han [21]	China	2015	2	SL+BP vs. SL	235 (2)	6 months	Recurrence; Healing time; Faecal Incontinence
He [22]	China	2009	2	SP vs. Fo	127 (4)	3 months	Healing time;
Ho [23]	Singapore	1998	2	Fo+Ms vs. Fo	103 (0)	3 months	Recurrence; Healing time; Faecal Incontinence
Ho [24]	Singapore	2001	2	SP vs. Fo	100 (8)	300 days	Healing time; Faecal Incontinence
Jain [25]	India	2012	2	Fo+Ms vs. Fe	40 (0)	12 weeks	Recurrence; Healing time; Faecal Incontinence
Koperen [26]	Netherlands	2011	2	AF vs. FP	60 (0)	120 days	Recurrence; Faecal Incontinence
Madbouly [27]	Egypt	2014	2	SL vs. AF	70 (0)	12 months	Recurrence; Faecal Incontinence
Mushaya [28]	Australia	2012	2	SL vs. AF	39 (2)	36 weeks	Recurrence; Healing time; Faecal Incontinence
Ortiz [29]	Spain	2009	2	AF vs. FP	42 (1)	12 months	Recurrence
Perez [30]	Spain	2003	2	Fo vs. AF	55 (0)	12 months	Recurrence; Healing time; Faecal Incontinence
Sahakitrungruang [31]	Thailand	2016	2	Fo+Ms vs. Fo	50 (0)	12 weeks	Recurrence; Faecal Incontinence
Wang [32]	China	2012	2	SP vs. Fo	60 (0)	12 months	Recurrence; Healing time; Faecal Incontinence
Zheng [33]	China	2015	2	SL+BP vs. SL	213 (26)	180 days	Recurrence;

BP = Biomaterial Plugging; AF = Advancement Flap; SP = Seton Placement; FG = Fibrin Glue; Fo = Fistulotomy; Fe = Fistulectomy; Ms = Marsupialisation; SL = Surgical Ligation; FP = Fistula Plug; LF = Loss to Follow-up;

### Fistulotomy plus marsupialisation exhibit the shortest healing time

**Figure 3:**
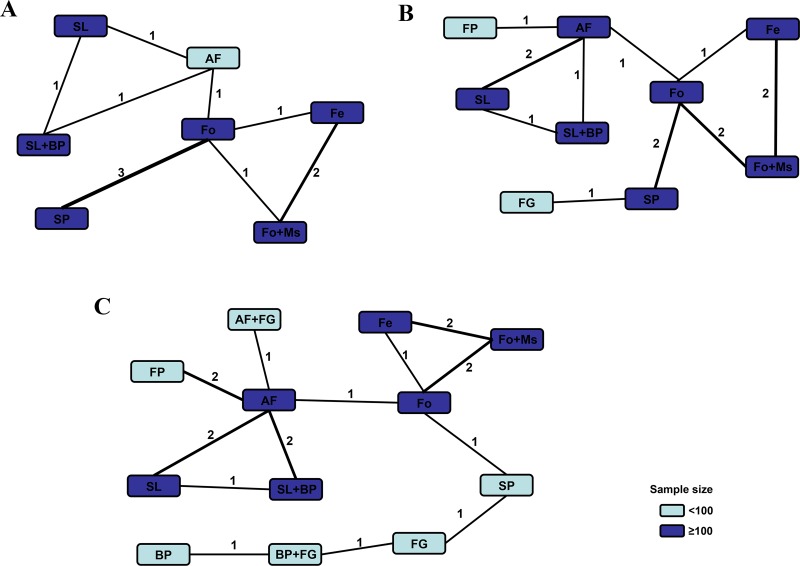
Comparison network of the included RCTs Each line connected 2 hemostatic strategies from the original studies. The number on the line represents the quality of studies comparing every pair of strategies. Study quality was also represented by the widths of the lines for included treatments regarding (**A**) healing time, (**B**) fecal incontinence and (**C**) recurrence.

**Figure 4:**
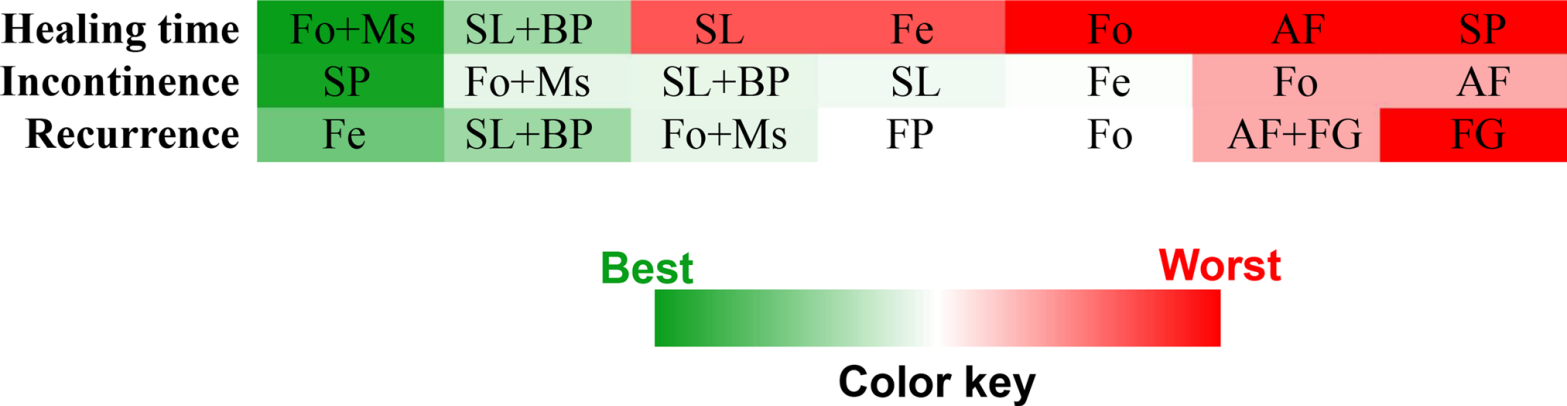
Ranks of different surgical treatments in terms of parameter-based *P* values

As presented in Table 1, 11 studies reported healing time data. We conducted a pooled estimation of all included surgical therapies and established a net connection (Figure 3A). After statistical comparison, we found that fistulotomy plus marsupialisation (Fo+Ms) demonstrated the shortest healing time (highest probability *P* = 0.69) compared with the other treatments (Supplementary Table 1) (Figure 4). Surgical Ligation plus Biomaterial Plugging (SL+BP) demonstrated the second highest value (*P* = 0.29).

### Seton placement is the best way to avoid postoperative fecal incontinence

Fourteen articles provided raw postoperative fecal incontinence data (Figure 3B). Network meta-analysis revealed that Seton Placement (SP) was the best technique to avoid postoperative incontinence, with the highest probability value of *P* = 0.66 (Supplementary Table 1) (Figure 4). Moreover, the Fo+Ms and SL+BP combination techniques both ranked in second place (probability *P* = 0.10, both), which meant they were suitable alternatives to reduce fecal incontinence rates if “Seton Placement” was not practicable.

### Fistulectomy provides the lowest recurrence rate

Interestingly, network comparison (Figure 3C) of 18 studies showed that “Fistulectomy” could be the best surgical therapy to reduce recurrence rates (Supplementary Table 1), (Figure 4) with a probability of 0.40. Furthermore, SL+BP revealed the second-best effect (*P* = 0.29) and Fo+Ms ranked third (*P* = 0.10) (Figure 4).

### Consistency and convergence analysis

After the overall quality of included studies was assessed, node-splitting models were conducted to assess inconsistency by testing for agreement between direct and indirect effects on a specific node (the split node). After constructing the node-splitting models, we observed that no significant inconsistencies were evident in this research (Table 2). The consistency model results were reliable (all *P* values were > 0.05). Furthermore, all parameter Potential Scale Reduction Factor (PSRF) values were limited to 1, which demonstrated that this research achieved good convergence efficiency.

**Table 2:** Results of node−splitting models

Item	Name	Odds Ratio (95% Confidence Interval)	Relative Effect (95% Confidence Interval)	Overall	*P*−Value
**Healing time**	AF, SL	−6.97 (−24.87, 9.82)	−9.02 (−34.57, 14.76)	−7.52 (−15.29, −0.26)	0.71
	AF, SL+BP	−17.07 (−34.25, 0.61)	−14.95 (−39.71, 10.07)	−16.40 (−23.99, −9.11)	0.68
	Fe, Fo	15.42 (1.48, 31.04)	20.16 (−2.04, 40.20)	17.75 (9.44, 25.61)	0.48
	Fe, Fo+Ms	−7.37 (−20.82, 4.44)	−12.11 (−31.46, 5.68)	−9.56 (−17.15, −1.05)	0.43
	Fo, Fo+Ms	−28.03 (−43.76, −12.77)	−23.16 (−46.40, −2.25)	−27.48 (−34.20, −18.61)	0.48
	SL, SL+BP	−8.00 (−24.51, 8.24)	−9.83 (−31.89, 13.73)	−8.87 (−16.67, −1.58)	0.71
**Faecal Incontinence**	AF, SL	−1.09 (−3.60, 0.89)	−2.71 (−51.19, 32.35)	−1.00 (−3.65, 0.90)	0.95
	AF, SL+BP	−8.23 (−49.34, 33.77)	−1.13 (−5.13, 2.56)	−0.94 (−5.13, 2.63)	0.70
	Fe, Fo	5.62 (−28.91, 52.64)	22.53 (−25.07, 63.19)	3.80 (−22.76, 34.34)	0.68
	Fe, Fo+Ms	−4.02 (−59.60, 35.32)	9.73 (−29.69, 43.80)	1.34 (−25.36, 32.06)	0.59
	Fo, Fo+Ms	−2.25 (−6.15, 0.52)	−16.11 (−75.36, 34.59)	−2.22 (−5.92, 0.53)	0.60
	SL, SL+BP	0.09 (−3.12, 3.07)	−16.96 (−54.49, 14.65)	0.16 (−3.05, 3.20)	0.36
**Recurrence**	AF, SL	−0.06 (−3.64, 3.51)	−21.18 (−81.35, 32.73)	−0.00 (−3.54, 3.58)	0.49
	AF, SL+BP	−2.36 (−7.43, 2.45)	−34.93 (−109.56, 31.73)	−2.40 (−7.15, 2.33)	0.39
	Fe, Fo	24.73 (−49.00, 111.87)	18.07 (−46.99, 107.94)	−3.13 (−61.29, 79.50)	0.94
	Fe, Fo+Ms	−12.62 (−95.16, 65.69)	12.76 (−59.53, 80.71)	−2.88 (−61.76, 79.27)	0.62
	Fo, Fo+Ms	−0.12 (−5.11, 5.00)	−57.50 (−188.25, 66.19)	−0.15 (−5.18, 4.90)	0.33
	SL, SL+BP	−26.69 (−116.70, 35.60)	−2.27 (−8.34, 3.71)	−2.41 (−8.20, 3.53)	0.52

## DISCUSSION

In this research, we aimed to conduct the first network meta-analysis to determine the best surgical treatment for anal fistulas. By comparing data based on 19 RCTs, our results revealed 3 different best treatments for 3 critical parameters with good consistency and convergence (Figure 4). Although these objective statistical results were understandable, they were also not final conclusions.

Fo+Ms was the best technique to reduce healing time. We know that most surgeons believed the classic fistulotomy technique was the correct therapy and the gold standard for treatment [30]. However, problems related to fistulotomy are numerous, including postoperative pain, bleeding and delayed wound healing [31]. The adverse effects of this technique could be changed if marsupialisation were performed simultaneously. We know that marsupialization of the open fistula was introduced to facilitate adequate lesion drainage, reduce healing time and improve continence by minimizing anal deformities [32]. Furthermore, the statistical results showed that Fo+Ms also ranked well in the other 2 parameters (Figure 4). Therefore, the clinical benefits of marsupialisation may be greater than previously thought.

Seton placement has been used for many years for treatment of anal fistulas. It was designed to prevent postoperative anal incontinence as the sphincter or soft tissue were slowly opened and both edges (or cross sections) became adherent due to the chronic shear stimulation of seton placement [33]. Furthermore, SP may facilitate drainage but also may impair wound healing and result in incomplete treatment. Accordingly, the results showed that while SP was the best way to avoid incontinence, it was useless for reducing healing time or recurrence rates (Figure 4).

Fistulectomy is a thorough treatment involving complete excision of the fistulous tracts, thereby providing complete tissue for histopathological examination and eliminating the risk of secondary tract formation [34]. With complete lesion removal, the recurrence rate is greatly reduced. However, a longer operating time and a longer healing process are required because the residual wound is wider than in other procedures and a higher incontinence rate is possible [35]. For these reasons, fistulectomy significantly reduced recurrence but did not affect the other parameters (Figure 4).

These finding have a number of potential clinical implications. First, SP and fistulectomy were objectively shown to be the best techniques to avoid fecal incontinence and reduce recurrence rates, respectively, although other surgical procedures continue to develop. The results also indicate that SP may cause the least damage due to its slow effects and limited localization. Thus, SP could maximize the retention of continence but would also delay healing time and increase recurrence rates. In contrast, fistulectomy is a direct and thorough procedure. SP completely removes lesions to reduce recurrence rates, although it is also associated with injuries to surrounding tissues. Accordingly, SP is unable to preserve sphincter function effectively. Because of these characteristics, the advantages of lesion resection and minimized local treatment seem contradictory. Is there a compromise or a better alternative? As our results show, Fo+Ms was most efficacious for reducing healing time, postoperative incontinence and recurrence rates. In comparison, fistulotomy performed much worse (Figure 4). Meanwhile, SL+BP also had good clinical efficacy compared to SL alone. Accordingly, we proposed the hypothesis that marsupialisation and biomaterial plugging had substantial clinical efficacy in their respective combination procedures. As mentioned above, a procedure with a limited range (such as SP) was not appropriate to reduce recurrence rates. However, extensive wounds may delay healing. Fistulotomy could expose a lesion and facilitate drainage to achieve complete treatment and reduce recurrence. Simultaneously, marsupialisation could result in more rapid healing [36]. Therefore, combined fistulotomy and marsupialisation may be an excellent choice for anal fistula patients. On the other hand, biomaterial plugging was found to be useful for its ability to suppress inflammation as well as potential differentiation [37]. However, biomaterial plugging alone could not provide significant clinical value [38]. Currently, biomaterial plugging is often used for surgical ligation to reduce local tissue damage as much as possible. Could a biological matrix be used for open wound surfaces to facilitate rapid recovery in the future? After comprehensive consideration, we may conclude that fistulotomy plus marsupialisation may be a superior surgical procedure for treatment of anal fistulas. On this basis, use of a biological matrix may provide even better clinical efficacy.

Notably, we discussed fistulotomy plus marsupialisation combined with biomaterials and demonstrated that they or their combination might be superior for surgical treatment of anal fistulas based on objective data. Nevertheless, we could not conclude that these procedures were suitable for every patient. Moreover, in certain respects, these procedures had several shortcomings. Our aim in this research was to provide directions for future surgical developments based on objective, evidence-based medical data. In summary, our results proved that extensive wound treatment procedures could increase incontinence rates and healing time, while procedures with limited range could increase recurrence rates. However, the combination of fistulotomy and marsupialisation could resolve this contradiction. Furthermore, combination with biomaterials may further improve the curative effects. Accordingly, we believe that an improved surgical technique of fistulotomy plus marsupialisation while exploiting multipurpose biomaterials may be the best direction for development in this field. Without a doubt, this conclusion needs to be verified in the future. Furthermore, we have to admit that certain significant limitations exist in this study. For instance, surgical procedures could be subjectively influenced by different surgeons in different nations. Patients with different habits and customs were also one of the confounding factors. Additionally, due to the content and sample sizes of included studies, subgroup analyses of different anal fistula classifications were not appropriate. Other factors such as operative time and blood loss were not included because we only included the parametric data that had the greatest impact on outcomes and we deemed some factors such as these to be less significant. Nevertheless, we aim to perform a more comprehensive literature review in the future.

Despite the existence of several limitations, our conclusion from this investigation is that fistulotomy plus marsupialisation demonstrated preliminary superior surgical utility for anal fistula treatment. Additionally, the combined use of biomaterials may provide better clinical efficacy. These options should be considered for future development in this field.

## MATERIALS AND METHODS

### Literature search and study selection

For inclusion in this study, research had to originate from studies that could be observed in globally recognized databases. The studies were not limited to certain languages, although full text had to be available for each study, and the English abstract had to be addressed. To avoid confounding factors associated with the development of surgical techniques, we only included publications from the last 20 years. Full consideration was given to a variety of conventional therapeutic approaches for anal fistula treatment from different studies which reached basic consensus or were demonstrated to be safe. Therefore, in this research, we only focused on the types of surgical strategies and objective parameters. Intraoperative details (intraoperative saline or antibiotic irrigation, for instance) were not primary considerations for surgical type classification. Additionally, comparisons based on improvements in the same surgical technique were not included in this research.

In accordance with PRISMA guidelines [39], an electronic search of PubMed, Embase and Cochrane Library was performed between June 1997 and June 2017. For a more comprehensive and inclusive review, we conducted an initial literature search of respective databases using only a few expressions such as “anal fistula (or archosyrinx)” and “randomized controlled trial”. Next, we expanded the search terms to include relevant topics to avoid neglecting eligible studies (an example of the search strategy in PubMed is listed in Supplementary Table 2). We included all germane human clinical research studies and we did not apply any language or publication status (e.g., online and offprint) restrictions. However, unpublished data or local databases were not searched. All retrieved articles with full text (including controversial papers) were reserved for final discussion concerning inclusion in this meta-analysis.

Inclusion criteria were as follows: (1) randomized controlled trials (RCTs); (2) at least one of the parameters including healing time, fecal incontinence and recurrence were provided in the studies; (3) raw data for continuous variables included means (and standard deviations) or medians (and ranges); and (4) each therapeutic surgical technique was performed as the only intervention.

Exclusion criteria eliminated studies with the following characteristics: (1) non-comparative studies; (2) incomplete raw data; (3) limitation to animals or cells; (4) data included neither means ± SD nor medians (ranges); (5) commentaries, review papers and articles with missing or unavailable data; (6) mixed or unclear treatment methods; or (7) research on Crohn's disease, malignant tumors or intestinal tuberculosis.

### Data extraction

For the full-text articles that were retrieved, 2 investigators (Qi W and Jun S) independently reviewed and checked the included studies to assess the available data and randomization. A predesigned electronic data abstraction form was used to extract relevant general information (e.g., authors and year of publication) and parametric data (e.g., study arms and sample sizes of each group). Data regarding healing time, fecal incontinence and recurrence rates were extracted.

### Quality assessment

Two reviewers (Qi W and Jun S) independently rated the study quality. The Cochrane risk of bias assessment tool [40] was used to assess the methodological quality of individual studies based on following criteria: whether the study was conducted with random sequence generation and allocation concealment (for selection bias assessment); whether blinding of participants and personnel occurred (for performance bias assessment); whether blinding of outcome assessment occurred (for detection bias assessment); whether incomplete outcome data were included (for attrition bias assessment); whether selective reporting was present (for reporting bias assessment) and whether the study exhibited other biases. All investigators (Qi W, Jun S and Yukun H) assessed the quality of the examined studies through discussion until reaching agreement.

### Statistical analyses

In this research, a comprehensive network meta-analysis based on the Bayesian theorem was necessary to compare every surgical treatment strategy for anal fistulas. This analysis method can be considered an extension of traditional pair-wise meta-analysis as it incorporates both direct and indirect information through a common comparator to estimate the relative interventional effects on the multiple intervention comparisons [41–42]. The 3 related parameters were considered to be pooled for comprehensive comparison. The different included paths had significant heterogeneity due to the completely different interventions. Accordingly, an initial random effects model was adopted for this study [43]. Summary measures were calculated as odds ratios (ORs) for dichotomous variables together with 95% confidence intervals (CIs), which were pooled for comprehensive comparison. We evaluated consistency by combining quantitative estimates from direct and indirect comparisons according to the experimental design and statistical data of included studies. If there were no relevant inconsistencies in the evidence, a consistency model could be used to reach conclusions about the effects of the included surgical treatments. A relevant rank probability plot (*P* value) could subsequently reveal the best therapeutic technique. A node-splitting analysis was also performed to demonstrate that no statistical inconsistencies existed when *P* > 0.05. Convergence was assessed to calculate the PSRF, in which values were limited to 1. In several included articles, data were presented in terms of medians and ranges. In these cases, the Hozo formula was used for data estimation and conversion [44]. Aggregate Data Drug Information System (ADDIS) automated software was used for network pooled estimation of all data analyses.

## SUPPLEMENTARY MATERIALS TABLES





## References

[1] Zanotti C, Martinez-Puente C, Pascual I, Pascual M, Herreros D, Garcia-Olmo D (2007). An assessment of the incidence of fstulain-ano in four countries of the European union. Int J Colorectal Dis.

[2] Marks CG, Ritchie JK (1977). Anal fstulas at St Mark's Hospital. Br J Surg.

[3] Li H, Jiang B, Yan J, Yang Z, Chen Y, Zhang W, Choy AC, Lee CY, Kang L (2011). A drug-laden elastomer for surgical treatment of anal fistula. Drug delivery and translational research.

[4] Zubaidi AM (2014). Anal fistula, Past and present. Saudi medical journal.

[5] Jacob TJ, Perakath B, Keighley MR (2010). Surgical intervention for anorectal fistula. Cochrane Database Syst Rev.

[6] Herreros MD, Garcia-Arranz M, Guadalajara H (2012). Autologous expanded adipose-derived stem cells for the treatment of complex cryptoglandular perianal fistulas: a phase III randomized clinical trial (FATT 1: fistula Advanced Therapy Trial 1) and long-term evaluation. Dis Colon Rectum.

[7] Pescatori M, Ayabaca SM, Cafaro D, Iannello A, Magrini S (2006). Marsupialization of fistulotomy and fistulectomy wounds improves healing and decreases bleeding: a randomized controlled trial. Colorectal Dis.

[8] Hjortrup A, Moesgaard F, Kjaergard J (1991). Fibrin adhesive in the treatment of perineal fistulae. Dis Colon Rectum.

[9] Limura E, Giordano P (2015). Modern management of anal fistula. World J Gastroenterol.

[10] A ba-bai-ke-re MM, Wen H, Huang HG, Chu H, Lu M, Chang ZS, Ai EH, Fan K (2010). Randomized controlled trial of minimally invasive surgery using acellular dermal matrix for complex anorectal fistula. World J Gastroenterol.

[11] Altomare DF, Greco VJ, Tricomi N, Arcanà F, Mancini S, Rinaldi M, Pulvirenti d'Urso A. (2011). Seton or glue for trans-sphincteric anal fistulae: a prospective randomized crossover clinical trial. Colorectal Dis.

[12] Chalya PL, Mabula JB (2013). Fistulectomy versus fistulotomy with marsupialisation in the treatment of low fistula-inano: a prospective randomized controlled trial. Tanzan J Health Res.

[13] Ellis CN, Clark S (2006). Fibrin Glue as an Adjunct to Flap Repair of Anal Fistulas: A Randomized, Controlled Study. Dis Colon Rectum.

[14] Filingeri V, Gravante G, Baldessari E, Casciani CU (2004). Radiofrequency fistulectomy vs. diathermic fistulotomy for submucosal fistulas: a randomized trial. Eur Rev Med Pharmacol Sci.

[15] Garcia-Olmo D, Herreros D, Pascual I, Pascual JA, Del-Valle E, Zorrilla J, De-La-Quintana P, Garcia-Arranz M, Pascual M (2009). Expanded adipose-derived stem cells for the treatment of complex perianal fistula: a phase II clinical trial. Dis Colon Rectum.

[16] Hammond TM, Porrett TR, Scott SM, Williams NS, Lunniss PJ (2011). Management of idiopathic anal fistula using cross-linked collagen: a prospective phase 1 study. Colorectal Dis.

[17] Han JG, Wang ZJ, Zheng Y, Chen CW, Wang XQ, Che XM, Song WL, Cui JJ (2016). Ligation of intersphincteric fistula tract vs ligation of the intersphincteric fistula tract plus a bioprosthetic anal fistula plug procedure in patients with transsphincteric anal fistula: early results of a multicenter prospective randomized trial. Ann Surg.

[18] He CM, Lu JG, Cao YQ, Yao YB (2009). [Design characteristics of clinical surgery trial based on treatment program of tunnel thread-drawing method for anal fistula: a prospective randomized controlled multicenter trial.] [Article in Chinese]. Zhong Xi Yi Jie He Xue Bao.

[19] Ho YH, Tan M, Leong AF, Seow-Choen F (1998). Marsupialization of fistulotomy wounds improves healing: a randomized controlled trial. Br J Surg.

[20] Ho KS, Tsang C, Seow-Choen F, Ho YH, Tang CL, Heah SM, Eu KW (2001). Prospective randomised trial comparing ayurvedic cutting seton and fistulotomy for low fistula-in-ano. Tech Coloproctol.

[21] Jain BK, Vaibhaw K, Garg PK, Gupta S, Mohanty D (2012). Comparison of a fistulectomy and a fistulotomy with marsupialization in the management of a simple anal fistula: a randomized, controlled pilot trial. J Korean Soc Coloproctol.

[22] Van Koperen PJ, Bemelman WA, Gerhards MF, Janssen LW, van Tets WF, van Dalsen AD, Slors JF (2011). The anal fistula plug treatment compared with the mucosal advancement flap for cryptoglandular high transsphincteric perianal fistula: a double-blinded multicenter randomized trial. Dis Colon Rectum.

[23] Madbouly KM, El Shazly W, Abbas KS, Hussein AM (2014). Ligation of intersphincteric fistula tract versus mucosal advancement flap in patients with high transsphincteric fistula-in-ano: a prospective randomized trial. Dis Colon Rectum.

[24] Mushaya C, Bartlett L, Schulze B, Ho YH (2012). Ligation of intersphincteric fistula tract compared with advancement flap for complex anorectal fistulas requiring initial seton drainage. Am J Surg.

[25] Ortiz H, Marzo J, Ciga MA, Oteiza F, Armendáriz P, de Miguel M (2009). Randomized clinical trial of anal fistula plug versus endorectal advancement flap for the treatment of high cryptoglandular fistula in ano. Br J Surg.

[26] Perez F, Arroyo A, Serrano P, Sánchez A, Candela F, Perez MT, Calpena R (2006). Randomized clinical and manometric study of advancement flap versus fistulotomy with sphincter reconstruction in the management of complex fistula-in-ano. Am J Surg.

[27] Sahakitrungruang C, Pattana-Arun J, Khomvilai S, Tantiphlachiva K, Atittharnsakul P, Rojanasakul A (2011). Marsupialization for simple fistula in ano: a randomized controlled trial. J Med Assoc Thai.

[28] Wang C, Lu JG, Cao YQ, Yao YB, Guo XT, Yin HQ (2012). Traditional Chinese surgical treatment for anal fstulae with secondary tracks and abscess. World J Gastroenterol.

[29] Zheng Y, Wang Z, Yang X, Cui J, Chen C, Zhang X, Wang X, Zhang X, Che X, Chen J, Cui F, Song W, Chen Y (2015). [A multicenter randomized controlled clinical trial of Ligation of the Intersphincteric Fistula Tract Plus Bioprosthetic Anal Fistula Plug in the treatment of chronic anal fistula.] [Article in Chinese]. Zhonghua Yi Xue Za Zhi.

[30] Göttgens KW, Janssen PT, Heemskerk J, van Dielen FM, Konsten JL, Lettinga T, Hoofwijk AG, Belgers HJ, Stassen LP, Breukink SO (2015). Long-term outcome of low perianal fistulas treated by fistulotomy: a multicenter study. Int J Colorectal Dis.

[31] Schouten WR, van Vroonhoven TJ (1991). Treatment of anorectal abscess with or without primary fistulectomy. Results of a prospective randomized trial. Dis Colon Rectum.

[32] Garcia-Aguilar J, Belmonte C, Wong WD, Goldberg SM, Madoff RD (1996). Anal fistula surgery. Factors associated with recurrence and incontinence. Dis Colon Rectum.

[33] Gewali MB, Pilapitiya U, Hattori M, Namba T (1990). Analysis of a thread used in the Kshara Sutra treatment in the Ayurvedic medicinal system. J Ethnopharmacol.

[34] Bhatt Y, Fatima S, Shaikh GS, Shaikh S (2011). Fistulotomy versus fistulectomy in the treatment of low fistula in ano. Rawal Medical Journal.

[35] (1996). Practice parameters for treatment of fistula-in-ano--supporting documentation. The Standards Practice Task Force. The American Society of Colon and Rectal Surgeons. Dis Colon Rectum.

[36] Malik AI, Nelson RL (2008). Surgical management of anal fistulae: a systematic review. Colorectal Dis.

[37] De Ugarte DA, Ashjian PH, Elbarbary A, Hedrick MH (2003). Future of fat as raw material for tissue regeneration. Ann Plast Surg.

[38] Champagne BJ, O'Connor LM, Ferguson M, Orangio GR, Schertzer ME, Armstrong DN (2006). Efficacy of anal fistula plug in closure of cryptoglandular fistulas: long-term follow-up. Dis Colon Rectum.

[39] Liberati A, Altman DG, Tetzlaff J, Mulrow C, Gøtzsche PC, Ioannidis JP, Clarke M, Devereaux PJ, Kleijnen J, Moher D (2009). The PRISMA statement for reporting systematic reviews and meta-analyses of studies that evaluate health care interventions: explanation and elaboration. J Clin Epidemiol.

[40] Higgins JPT, Green S (2011). Cochrane handbook for systematic reviews of interventions. The Cochrane Collaboration.

[41] Salanti G, Higgins JP, Ades AE, Ioannidis JP (2008). Evaluation of networks of randomized trials. Stat Methods Med Res.

[42] Jansen JP, Crawford B, Bergman G, Stam W (2008). Bayesian meta-analysis of multiple treatment comparisons: an introduction to mixed treatment comparisons. Value Health.

[43] Lu G, Ades A (2012). Assessing evidence inconsistency in mixed treatment comparisons. J Am Statist Assoc.

[44] Hozo SP, Djulbegovic B, Hozo I (2005). Estimating the mean and variance from the median, range, and the size of a sample. BMC Med Res Methodol.

