# Supplementary material for: The best surgical strategy for anal fistula based on a network meta-analysis
Source: Oncotarget. 2017 Oct 12;8(58):99075–84. doi: 10.18632/oncotarget.21836 (PMC5716793; doi:10.18632/oncotarget.21836)
Supplement: Supplementary file 1 [file oncotarget-08-99075-s001.pdf]

The best surgical strategy for anal fistula based on a network meta-analysis

SUPPLEMENTARY MATERIALS

Supplementary Table 1: Search strategy in pubmed

Pubmed
#1 anal fistula OR archosyrinx
#2 cure time ORhealing timeOR wound healing
#3 incontinence OR irretention
#4 palindromia OR recrudescence OR recurrence
#5 randomized controlled trial
#6 #1 AND #2 AND #5
#7 #1 AND #3 AND #5
#8 #1 AND #4 AND #5
#9 #6 AND #7 AND #8

Supplementary Table 2: The network meta-analysis results for different surgical treatments. See Supplementary_Table_2
